# Alleviating effect of lavender (*Lavandula angustifolia*) and its major components on postherpetic pain: a randomized blinded controlled trial

**DOI:** 10.1186/s12906-024-04362-z

**Published:** 2024-01-24

**Authors:** Jiyeong You, You Kyoung Shin, Geun Hee Seol

**Affiliations:** 1 Department of Basic Nursing Science, College of Nursing, Korea University, 145 Anam-ro, Seongbuk-gu, Seoul, 02841 Republic of Korea; 2 BK21 FOUR Program of Transdisciplinary Major in Learning Health Systems, Graduate School, Korea University, Seoul, Republic of Korea

**Keywords:** Complementary and alternative medicine, Lavender, Linalool, Linalyl acetate, Postherpetic neuralgia

## Abstract

**Background:**

Postherpetic neuralgia (PHN) causes severe pain which can lead to decreased quality-of-life. This study aimed to evaluate the effects of inhalation of lavender (*Lavandula angustifolia*) oil and its major components (linalool and linalyl acetate) on the pain in patients with PHN.

**Methods:**

This study was performed at an outpatient clinic. Sixty-four patients with postherpetic neuralgia were randomly allocated to a control group (almond oil) or one of three experimental groups (lavender oil, linalool, or linalyl acetate diluted in almond oil at concentration of 1% v/v), and the participants inhaled the aroma by natural breathing. Quality, severity, and intensity of pain were measured before and after the intervention.

**Results:**

Six patients discontinued the intervention for personal reasons; hence, data from 58 patients were analyzed (control group, *n* = 14; 1% lavender oil group, *n* = 15; 1% linalool, *n* = 15; 1% linalyl acetate, *n* = 14). Reduction in sensory pain was greater in the 1% lavender oil group, 1% linalool group, and 1% linalyl acetate group than in the control group (all *P* < 0.001). Reduction in affective pain was greater in the 1% lavender group (*P* < 0.001) and the 1% linalool group (*P* = 0.007) than in the control group. Decreases in pain severity and intensity were significantly greater in all three intervention groups than in the control group.

**Conclusions:**

Inhalation of lavender oil and its major volatile components effectively reduced the quality, severity, and intensity of postherpetic pain, suggesting that lavender oil, linalool, and linalyl acetate may each be an effective intervention for reducing pain in patients with postherpetic neuralgia.

**Trial registration:**

This study was retrospectively registered on the Clinical Research Information Service. Registration number: KCT0007772, first registration 06/10/2022.

## Background

Postherpetic neuralgia is a persistent neuropathic pain induced by the reactivation of the varicella zoster virus [1]. The lifetime risk of herpes zoster is approximately 30% [2], and the incidence rate of postherpetic neuralgia after herpes zoster ranges from 5 to 30%, depending on patient age [3]. Patients with this condition often describe their pain as a constant burning that can be accompanied with hyperalgesia and allodynia [4]. Chronic pain is related to sleep disturbances, depression, and anxiety and poor quality-of-life [5]. Moreover, complications related to herpes zoster, including postherpetic neuralgia, can increase direct healthcare costs [6]. Considering the detrimental impact of postherpetic neuralgia on patients and society, its management should be a priority for healthcare workers.

The current options for treating postherpetic neuralgia include tricyclic antidepressants (TCAs), antiepileptic drugs, opioids, tramadol, lidocaine, and capsaicin patches [1]. However, patients must take these medications for a long time, thus increasing the risk of adverse effects. For example, TCAs can cause anticholinergic toxicity [7]; opioids can lead to nausea, vomiting, respiratory depression, constipation, sedation and dependency [8]; and lidocaine patches can cause skin irritation, skin redness and rash [9]. Moreover, the pain relief provided by pharmacotherapy is often limited [10]. Given the above, it is necessary to find an intervention that has high efficacy and leads to few adverse effects.

Aromatherapy uses essential oils extracted from stems, leaves, flowers, fruits, and roots. Inhalation of aromatic scents stimulates the limbic system in the brain. In addition, inhaled scents enter into the capillary blood vessels through the alveoli, thereby affecting the endocrine and autonomic nervous systems [11]. Therefore, aromatic scents can provide benefits in various clinical settings [12]. For example, inhalation of bergamot oil decreased pain in patients who received surgery for lumbar spinal stenosis [13]. A study of mice reported that eucalyptus oil had an antinociceptive effect on visceral pain induced by acetic acid treatment [14]. A meta-analysis showed that aromatherapy was effective in reducing pain and caused no adverse effects [15]. These previous studies thus indicate that aromatherapy has potential for the clinical management of pain.

Lavender (*Lavandula angustifolia*) oil, which is commonly used in perfumery, cosmetics, and pharmaceuticals, has two major volatile components — linalool and linalyl acetate [16]. Inhaled volatile compounds act rapidly *via* the limbic system, and this is believed to be responsible for their beneficial biological effects [17]. A study of mice with neuropathic pain reported that oral administration of lavender oil led to antihyperalgesic effects and decreased the phosphorylation of mitogen-activated protein kinases in the spinal cord [18]. Another study of mice with neuropathic pain induced by spinal nerve ligation reported that linalool reduced mechanical allodynia [19]. A clinical study of patients who received colorectal cancer surgery reported that inhalation of lavender oil or linalyl acetate following removal of an indwelling urinary catheter significantly decreased urinary residual sense and pain scores [20].

The above findings suggest that lavender oil and its main compounds could possibly reduce the severity and intensity of pain in patients with postherpetic neuralgia, but this topic has not yet been examined. Therefore, the purpose of the present study was to investigate the effects of inhalation of lavender oil and its main components (linalool and linalyl acetate) on the quality, severity, and intensity of pain in patients with postherpetic neuralgia.

## Methods

### Study design and participants

This randomized controlled trial had a pretest-posttest design and was performed in accordance with the seventh revision (2017) of the Declaration of Helsinki. All research procedures were approved by the Institutional Review Board of Korea University Ansan Hospital (institutional review board No. AS12200), and this study was registered on the Clinical Research Information Service (Registration number: KCT0007772).

The participants of this study were patients diagnosed with postherpetic neuralgia who visited the outpatient clinic at a university hospital in Korea. Eligible participants were informed of the procedures and purposes of this study, and all of them voluntarily provided written informed consent. There was no age limit for study participants. Also, participants were included if they did not receive a previous neurological treatment for herpes zoster, and were conscious and able to communicate. Patients with postherpetic neuralgia who have mild pain can be treated with nonsteroidal agents or acetaminophen. By contrast, patients with moderate to severe pain need to be managed with opioids, which can lead to multiple adverse effects [21]. Therefore, the present study included only participants who had a visual analogue scale (VAS) pain score of 4 or greater (moderate to severe pain) [22]. Participants were excluded if they had olfactory dysfunction, allergic reaction to the aroma, were taking medications due to a psychiatric illness, or were receiving hormone therapy or aromatherapy.

Based on a statistical power of 0.70, a significance level of 0.05, and an effect size of 0.40 determined in a previous study [23], the G*Power program determined the minimum sample size was 60 patients. The statistical power chosen for this study, 0.70, is comfortably within the range of values currently accepted as providing sufficient power [24]. Also, the chosen effect size of 0.40 has been described as large in contexts where there are many means to be compared [25]. Based on attrition, 64 participants were randomly assigned to the control or experimental groups by using a simple random assignment method. The random allocation sequence was generated using Random Allocation Software (version 2.0). The random allocation sequence was generated by an independent researcher, and the allocation sequence was concealed from the researcher who recruited participants.

### Measurements

The short form of the McGill Pain Questionnaire (SF-MPQ) was developed to measure three components of pain: quality, severity, and intensity [26]. A previous study used the Korean version of the SF-MPQ to assess the quality, severity, and intensity of pain [27]. Cronbach’s α for this questionnaire was 0.93 in a previous study [27] and 0.84 in the present study.

The first 15 descriptors of this metric (4 affective and 11 sensory) assess the quality of pain. Each descriptor was rated on a 4-point Likert scale (0 = ‘none’, 1 = ‘mild’, 2 = ‘moderate’, 3 = ‘severe’). A higher score indicated a higher pain level.

The severity of pain was assessed using a VAS. This scale consisted of a straight horizontal line of 100 mm that was labeled 0 (no pain) on the leftmost end and 10 (maximum pain imaginable) on the rightmost end. Participants were asked to mark the severity of pain on this line. A higher score indicated more severe pain.

The intensity of pain was measured using a 6-point present pain intensity (PPI) Likert scale (0 = ‘no pain’, 1 = ‘mild’, 2 = ‘discomfort’, 3 = ‘distressing’, 4 = ‘horrible’, 5 = ‘excruciating’). A higher score indicated a higher intensity of pain.

The level of depression was evaluated using the Beck Depression Inventory (BDI), a metric that has 21 items covering cognitive, emotional, motivational, and physiological domains [28]. A previous study reported that the Korean version of the BDI was reliable and valid [29]. Each item in the BDI contains 4 statements regarding severity, and participants were asked to choose the statement closest to how they feel. Each answer was scored from 0 to 3 points, and the total score ranged from 0 to 63 points. A higher score indicated a higher level of depression. Cronbach’s α for the Korean version of the BDI was 0.88 in a previous study [29]. Psychological factors such as depression are related to the development of chronic pain [30]. Moreover, a previous study reported that fluoxetine has greater analgesic efficacy in depressive patients than in non-depressive patients [31], suggesting that the analgesic effects of this agent may vary depending on whether the patient has depression. Therefore, the present study measured the level of depression using the BDI, with the findings ruling out the possibility that the level of depression affected the pain experienced by participants.

### Intervention

Preliminary testing was performed to determine the optimal concentration of lavender essential oil, linalool, and linalyl acetate. Three healthy adults who agreed to participate in this preliminary investigation inhaled aroma consisting of 1%, 3%, or 5% (v/v) lavender oil (Aromarant Co., Rottingen, Germany), linalool (Sigma-Aldrich, MO, USA), or linalyl acetate (Sigma-Aldrich, MO, USA) in almond oil. The results indicated the optimal concentration for each of the agents was 1%, a level that was not irritating and that lasted until the end of the intervention (results not shown).

Lavender (*Lavandula angustifolia*) oil and almond oil were purchased from Aromarant Co. (Rottingen, Germany), and linalool and linalyl acetate were purchased from the Sigma-Aldrich (MO, USA). Lavender oil, linalool or linalyl acetate was diluted in almond oil at a concentration of 1% (v/v) based on the preliminary tests. Almond oil, 1% lavender essential oil, 1% linalool, and 1% linalyl acetate were prepared in identical bottles and stored in a refrigerator at 4 ℃. Eligible participants completed questionnaires that assessed their general characteristics, and also completed the SF-MPQ and BDI before the intervention. After the survey, participants were allowed to rest for 5 min in a seated position. A curtain was then drawn to obscure experimental cues. For each experimental group, 1 mL of 1% lavender essential oil, 1% linalool, or 1% linalyl acetate was applied to a piece of gauze that was placed under the individual’s nose. For the control group, pure almond oil was applied to a piece of gauze that was placed under the participant’s nose. A previous study found that inhalation of an essential oil for 5 min was effective in reducing severity of pain in patients before selective nerve root block [23]. In addition, the maximum levels of a lavender monoterpenoid were detectable in the blood stream within 20 min after inhalation [32]. Therefore, participants inhaled the 1% lavender oil, 1% linalool, or 1% linalyl acetate (experimental) or almond oil (control) with natural breathing for 5 min, and then rested for 15 min so that the inhaled volatile odorant molecules could diffuse sufficiently into the circulation. After that, the post-test was performed in exactly the same manner as the pre-test. The intervention was conducted in the same room for all patients to control for the presence of other odors, and the room was sufficiently ventilated to prevent remaining scent from affecting the next participant. All participants were blinded to the type and possible efficacy of the fragrance. To reduce bias, a single independent researcher performed the preparation and administration of lavender oil, linalool, linalyl acetate, and almond oil. The researcher who prepared and administered the fragrance was not blinded to group allocation. However, the assessor was blinded to group assignment, and all assessment procedures were conducted by the same researcher in the same place to ensure measurement consistency.

### Statistical analysis

All data were analyzed using SPSS version 24.0 software (IBM, NY, USA). To evaluate the homogeneity of the four groups, categorical variables were analyzed by Pearson’s Chi-squared test or Fisher’s exact test. Specifically, Fisher’s exact test was used according to expected frequencies [33]. The Shapiro-Wilk test was used to test normality. Continuous variables were analyzed by one-way analysis of variance when the data were normally distributed, and the Kruskal-Wallis rank sum test was used when the data were not normally distributed [34]. Differences in outcome variables among the four groups were analyzed using Kruskal-Wallis rank sum test, followed by a *post hoc* Mann-Whitney test because the data did not meet the normality condition. Also, differences in outcome variables within a group were analyzed using a paired *t*-test when the data were normally distributed, and the Wilcoxon signed-rank test was used when the data were not normally distributed [34]. A *P* value less than 0.05 was considered statistically significant.

## Results

### General characteristics and homogeneity testing

**Fig. 1:**
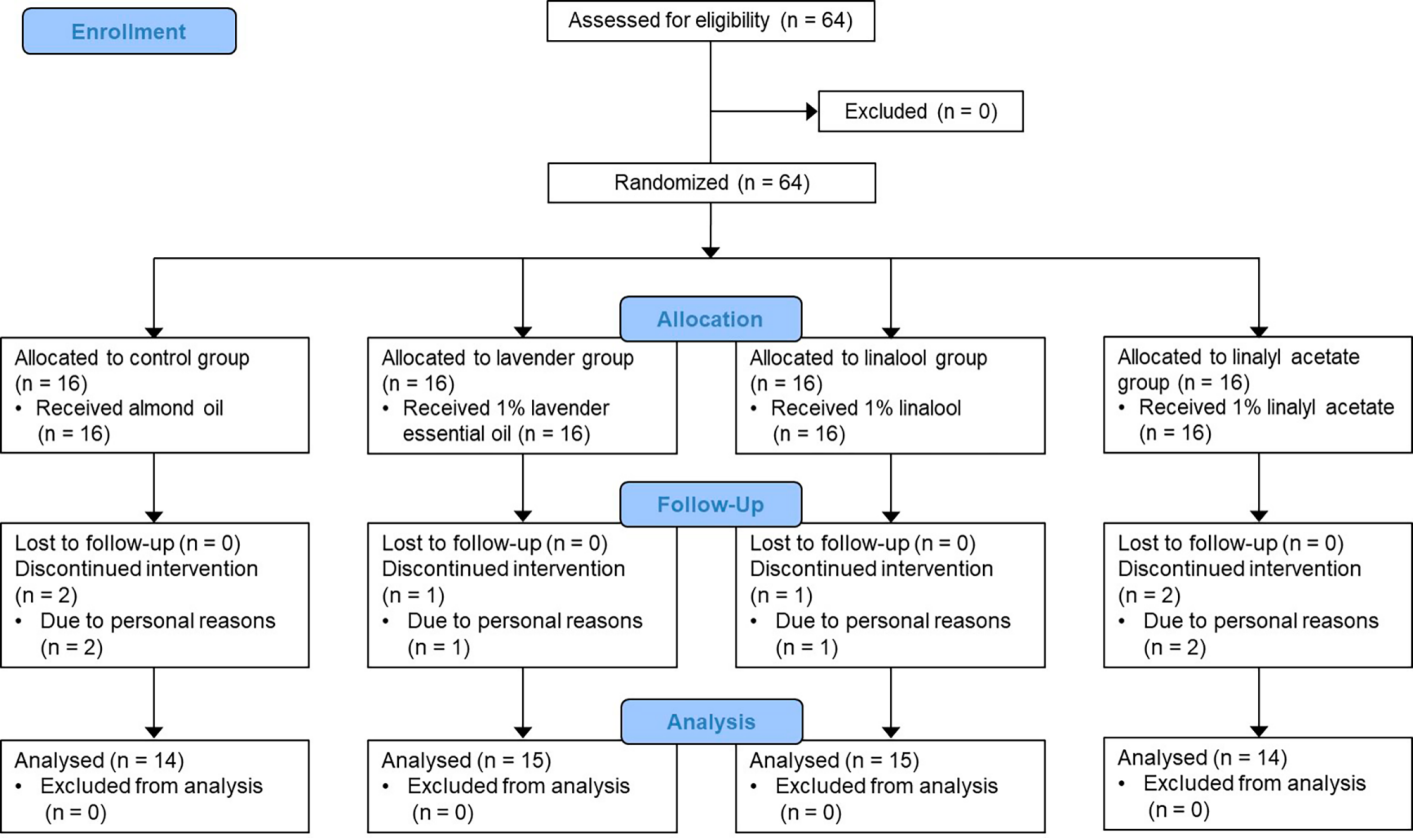
CONSORT flow diagram of study design

Six of the 64 patients dropped out for personal reasons, such that 58 participants were included in the final analysis (Fig. 1). There were 35 females (60.3%) and 23 males (39.7%), the mean age was 64.09 ± 15.27 years, and the average duration of pain after diagnosis was 10.79 ± 9.46 weeks. The thoracic dermatome (46.5%) was the most common site for development of herpes zoster, followed by the lumbar (20.7%), trigeminal (19.0%), and cervical (13.8%) dermatomes. Rash severity was mild in 21 patients (36.2%), moderate in 30 patients (51.7%), and severe in 7 patients (12.1%). Most patients received treatment with anticonvulsants (94.8%) and/or analgesics (86.2%). Hypertension (44.8%) was the most common comorbidity, followed by diabetes mellitus, tuberculosis, and hepatitis. The average BDI score was 10.45 ± 6.91. The four groups had no significant differences in general characteristics (Table 1). The outcome variables were measured using the SF-MPQ, and the four groups were similar in terms of pain characteristics (Table 2).

**Table 1:** Demographic and clinical characteristics of enrolled participants

Variable		Control (*n* = 14)	1% Lavender (*n* = 15)		1% Linalool (*n* = 15)	1% Linalyl acetate (*n* = 14)	*P* value
Gender							
	Male	6 (42.9)	6 (40.0)		5 (33.3)	6 (42.9)	0.946†
	Female	8 (57.1)	9 (60.0)		10 (66.7)	8 (57.1)	
Age (years)		68.50 (52.50–79.50)	69.00 (59.00–79.00)		62.00 (49.00–70.00)	58.50 (51.75-79.00)	0.826‡
Pain duration (weeks)		8.00 (6.75–13.75)	5.00 (5.00–12.00)		7.00 (4.00–13.00)	7.00 (4.00–21.00)	0.645‡
Dermatomal distribution							
	Trigeminal	2 (14.3)	4 (26.7)		3 (20.1)	2 (14.3)	0.939
	Cervical	2 (14.3)	2 (13.3)		2 (13.3)	2 (14.3)	
	Thoracic	8 (57.1)	6 (40.0)		5 (33.3)	8 (57.1)	
	Lumbar	2 (14.3)	3 (20.0)		5 (33.3)	2 (14.3)	
Rash severity							
	Mild (< 25 lesions)	4 (28.6)	7 (46.7)		5 (33.3)	5 (35.7)	0.767
	Moderate (25–50 lesions)	9 (64.3)	5 (33.3)		8 (53.3)	8 (57.1)	
	Severe (> 50 lesions)	1 (7.1)	3 (20.0)		2 (13.3)	1 (7.1)	
Medications							
	Tricyclic antidepressant	11 (78.6)	7 (46.7)		9 (60.0)	6 (42.9)	0.212†
	Anticonvulsant	14 (100.0)	14 (93.3)		13 (86.7)	14 (100.0)	0.605
	Analgesic	11 (78.6)	14 (93.3)		12 (80.0)	13 (92.9)	0.578
	5% lidocaine patch	3 (21.4)	2 (13.3)		3 (20.0)	2 (14.3)	0.967
Hypertension							
	Yes	4 (28.6)	8 (53.3)		9 (60.0)	5 (35.7)	0.284†
	No	10 (71.4)	7 (46.7)		6 (40.0)	9 (64.3)	
Diabetes mellitus							
	Yes	1 (7.1)	2 (13.3)		4 (26.7)	1 (7.1)	0.490
	No	13 (92.9)	13 (86.7)		11 (73.3)	13 (92.9)	
Hepatitis							
	Yes	0 (0.0)	1 (6.7)		0 (0.0)	1 (7.1)	0.864
	No	14 (100.0)	14 (93.3)		15 (100.0)	13 (92.9)	
Tuberculosis							
	Yes	0 (0.0)	0 (0.0)		2 (13.3)	1 (7.1)	0.503
	No	14 (100.0)	15 (100.0)		13 (86.7)	13 (92.9)	
BDI score		8.21 ± 4.96	12.13 ± 8.12		11.73 ± 7.61	9.50 ± 6.32	0.379^§^

Data are reported as n (%), mean ± standard deviation or median (interquartile range)

Data are analyzed using Fisher’s exact test unless otherwise indicated

^†^Pearson’s Chi-squared test; ^‡^Kruskal-Wallis rank sum test; ^§^One-way ANOVA

BDI = Beck Depression Inventory

**Table 2:** Homogeneity tests for SF-MPQ parameters

SF-MPQ parameter	Control (*n* = 14)	1% Lavender (*n* = 15)	1% Linalool (*n* = 15)	1% Linalyl acetate (*n* = 14)	*P* value
Sensory (score)	10.07 ± 4.34	14.73 ± 7.30	13.07 ± 7.12	14.36 ± 5.90	0.202
Affective (score)	3.00 (1.00–5.00)	4.00 (4.00–8.00)	3.00 (2.00–7.00)	2.50 (1.00-5.25)	0.072†
Total (score)	13.21 ± 5.89	20.27 ± 9.58	17.27 ± 9.32	17.50 ± 7.55	0.163
VAS (mm)	58.86 ± 12.43	65.20 ± 15.34	63.13 ± 19.78	63.64 ± 15.15	0.745
PPI (score)	2.00 (2.00–3.00)	3.00 (2.00–4.00)	2.00 (2.00–3.00)	3.00 (2.00–4.00)	0.099†

Data are reported as mean ± standard deviation or median (interquartile range)

Data are analyzed using one-way ANOVA unless otherwise indicated

^†^Kruskal-Wallis rank sum test was applied because the data did not satisfy the normality condition

PPI = present pain intensity; SF-MPQ = Short Form of the McGill Pain Questionnaire; VAS = visual analogue scale

### Effects of lavender oil and its major components on quality of pain

**Fig. 2:**
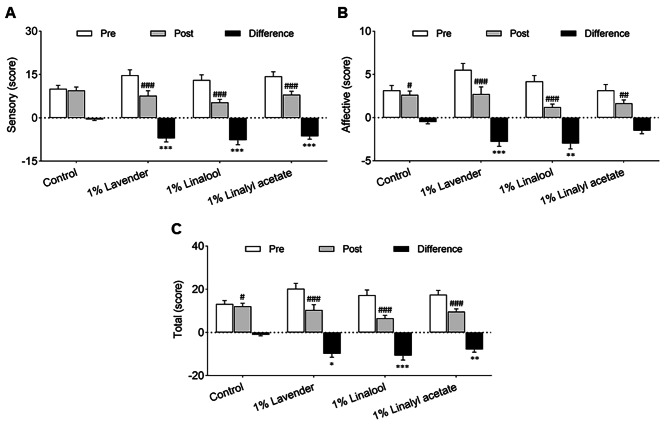
Effects of inhalation of lavender oil and its major components (linalool and linalyl acetate) on the quality of pain in patients with postherpetic neuralgia. (**A)** Sensory score, **(B)** Affective score, **(C)** Total score. Results are presented as mean ± standard error of the mean. ^#^*P* < 0.05, ^##^*P* < 0.01, ^###^*P* < 0.001 vs. Pretreatment; ^**^P < 0.01, ^***^P < 0.001 vs. Control

This study used the SF-MPQ to measure sensory pain, affective pain, and total pain before and after treatment (Fig. 2A-C). The reduction in sensory pain was significantly greater in the 1% lavender group (− 7.07 ± 1.30 score, *P* < 0.001), 1% linalool group (− 7.73 ± 1.59 score, *P* < 0.001), and 1% linalyl acetate group (− 6.36 ± 1.03 score, *P* < 0.001) than in the control group (− 0.57 ± 0.36 score). The reduction in affective pain was also significantly greater in the 1% lavender group (− 2.80 ± 0.53 score, *P* < 0.001) and 1% linalool group (− 3.00 ± 0.62 score, *P* = 0.007) than in the control group (− 0.50 ± 0.23). The affective pain reduction tended to be greater in the 1% linalyl acetate group than in the control group (*P* = 0.092), but this difference did not reach statistical significance. The reduction in total pain score was significantly greater in all three intervention groups (all *P* < 0.05) than in the control group.

### Effects of lavender oil and its major components on severity of pain

**Fig. 3:**
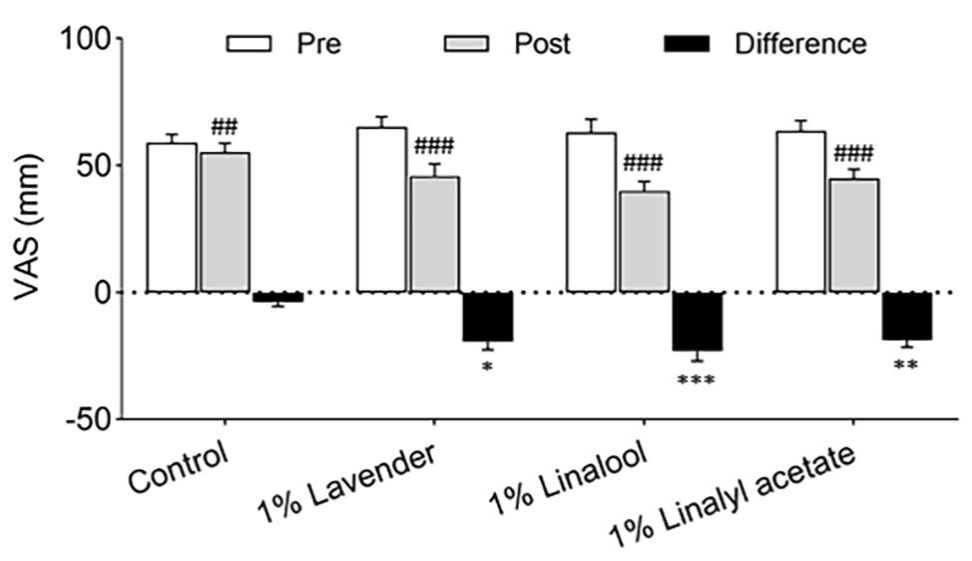
Effects of inhalation of lavender oil and its major components (linalool and linalyl acetate) on the severity of pain in patients with postherpetic neuralgia. Results are presented as mean ± standard error of the mean. ^##^*P* < 0.01, ^###^*P* < 0.001 vs. Pretreatment. ^*^P < 0.05, ^**^P < 0.01, ^***^P < 0.001 vs. Control. VAS = visual analogue scale

This study used a VAS to evaluate the severity of pain (Fig. 3). The results showed a significant decrease of pain in the 1% lavender group (− 19.40 ± 3.26 mm, *P* = 0.011), 1% linalool group (− 23.20 ± 3.92 mm, *P* < 0.001), and 1% linalyl acetate group (− 18.64 ± 2.95 mm, *P* = 0.008) compared with the control group (− 3.64 ± 1.81 mm).

### Effects of lavender oil and its major components on intensity of pain

**Fig. 4:**
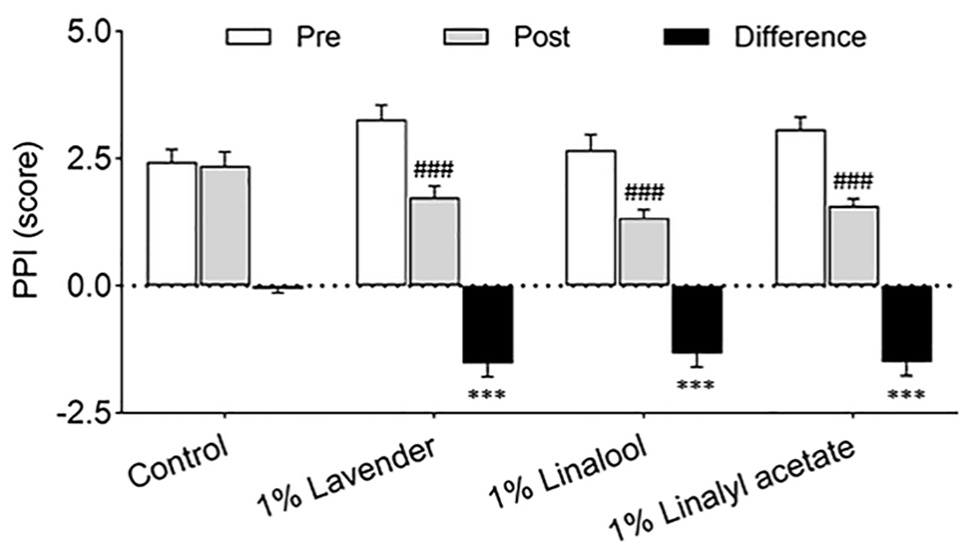
Effects of inhalation of lavender oil and its major components (linalool and linalyl acetate) on the intensity of pain in patients with postherpetic neuralgia. Results are presented as Results are presented as mean ± standard error of the mean. ^###^*P* < 0.001 vs. Pretreatment. ^***^P < 0.001 vs. Control. PPI = present pain intensity

This study also used a 6-point PPI Likert scale to assess the intensity of pain (Fig. 4). The results showed a significant decrease of pain in the 1% lavender group (− 1.53 ± 0.26 score, *P* < 0.001), 1% linalool group (− 1.33 ± 0.27 score, *P* < 0.001), and 1% linalyl acetate group (− 1.50 ± 0.27 score, *P* < 0.001) compared with the control group (− 0.07 ± 0.07 score).

## Discussion

There is increasing interest in complementary and alternative medicine using natural products. For example, 36.0% of patients with chronic disease in Thailand indicated that they had used herbal medicine within the past 12 months [35]. Treatment with herbs was the preferred type of complementary and alternative medicine in patients living with human immunodeficiency virus [36] and hypertension [37]. Also, 42% of physicians in Italy believed that complementary and alternative medicine could play an integrative role in conventional medicine [38]. Aromatherapy is used as a complement to traditional care and alternative medicine, and can be beneficial for managing symptoms such as pain [12]. In line with these trends, the present study examined the pain-reducing effects of lavender oil and its major components (linalool and linalyl acetate) in patients with postherpetic neuralgia. The SF-MPQ was used to quantify the sensory and affective components of pain, and the severity and intensity of pain.

The results demonstrated that the sensory dimension of pain was significantly reduced by inhalation of lavender essential oil, linalool, or linalyl acetate, but that almond oil (control) had no effect. Sensory pain can be classified as stabbing pain or heavy pain, and this two-factor model is in line with distinctive pain sensations mediated by Aδ and C nerve fibers [39]. A previous study of mice showed that application of linalool or linalyl acetate inhibited the nociceptive behavioral response to capsaicin [40], a treatment that induces hyperalgesia by activating the C nerve fibers [41]. Another study of mice showed that linalyl acetate decreased the nerve injury induced-pain behaviors and thymic stromal lymphopoietin production in Aδ and C nerve fibers [42]. These findings suggest a possible mechanism by which lavender oil or its main components (linalool and linalyl acetate) reduce the stabbing pain and heavy pain mediated by Aδ and C nerve fibers in patients who have postherpetic neuralgia.

The common descriptors of the affective dimension of pain include “tiring-exhausting”, “sickening”, “fearful”, and “punishing-cruel”. Chronic pain, including postherpetic neuralgia, is frequently accompanied by fatigue, depression, anxiety, and reduced quality-of-life [43]. For example, approximately one-third of patients with postherpetic neuralgia reported emotional problems, stress, or depression [44]. The present study found that inhalation of lavender oil or linalool significantly decreased the affective dimension of pain; linalyl acetate also tended to reduce this pain, but the effect was not statistically significant. A previous clinical study of patients with diabetes showed that massage with lavender oil reduced neuropathic pain and improved quality-of-life [45]. Another clinical study found that inhalation of lavender oil by community-dwelling older people significantly decreased their levels of anxiety, depression, and stress [46]. A study of rats found that inhalation of lavender oil ameliorated anxiety-like and depression-like behaviors induced by corticosterone injection [47]. A clinical study showed that inhalation of linalyl acetate reduced the levels of anxiety and stress in cancer patients prior to chemotherapy [48]. The present finding that lavender oil reduced affective pain in patients with postherpetic neuralgia (Fig. 2) is consistent with many previous results. Therefore, the effects of lavender oil in reducing affective pain in patients with postherpetic neuralgia may be due to its anti-anxiety, anti-depressant, and anti-stress properties.

In the present study, patients who inhaled lavender essential oil, linalool, or linalyl acetate had significantly reduced severity and intensity of pain. These results are in agreement with recent studies that observed that inhalation therapy and massage therapy using lavender oil reduced the severity of labor pain in primiparous women [49], and that inhalation of lavender oil decreased the intensity of labor pain in primiparous women [50]. Similarly, another study of patients who underwent removal of indwelling urinary catheters after colorectal cancer surgery reported that the VAS pain score was decreased after inhalation of lavender oil or linalyl acetate [20]. Postherpetic neuralgia is characterized by γ-aminobutyric acid (GABA) interneuron deficits, and increased glutamate concentrations and sympathetic activation [51]. Previous research showed that linalool enhanced GABA-evoked currents of human α_1_β_2_ GABA_A_ receptors [52] and protected neuronal cells from oxidative stress induced by glutamate [53]. Also, a study of rats with acute nicotine exposure showed that linalyl acetate led to a significant recovery of heart rate, possibly by balancing the autonomic nervous system [54]. The findings of these previous studies are consistent with those of the present study, suggesting that the reductions in pain severity and intensity observed after inhalation of lavender essential oil, linalool, or linalyl acetate may be related to homeostasis of GABA-evoked currents, protection from increased glutamate levels, and balance in the autonomic nervous system.

The key innovative contribution of the present study was the finding that administering lavender essential oil, linalool or linalyl acetate to patients with postherpetic neuralgia who were already taking conventional pain medications decreased pain levels even further, with a low risk of adverse effects. Specifically, the patients included in this study took medications including TCAs, anticonvulsants, and lidocaine patches to reduce pain. The results showed that these patients received further pain relief by additionally inhaling lavender essential oil, linalool or linalyl acetate. The effects of these agents in patients with postherpetic neuralgia taking pain medications are supported by previous findings showing that postoperative demand for opioids was significantly lower in patients who inhaled lavender oil than in the patients who received placebo [55], and that the antinociceptive effect of morphine was significantly enhanced by combined treatment with linalool in mice with capsaicin-induced pain [40]. Therefore, these findings present that inhalation of lavender oil, linalool or linalyl acetate was effective in reducing pain in patients with postherpetic neuralgia who were already taking conventional pain medications.

### Limitations

The present study had some limitations. First, the study was conducted in a single institution and included a relatively small number of patients. The small sample size may have been due to the relatively low value of 0.7 used for the statistical power. Therefore, there is a need for further experiments conducted in multiple centers with a large sample size. Second, the mean age of participants was 64.09 years in the present study, indicating that the findings of the present study cannot be easily generalized to young adults with postherpetic neuralgia, especially given previous results showing that older adults have a significantly higher pain threshold than young adults [56]. Therefore, further studies are needed to assess the effects of inhalation of lavender essential oil, linalool, or linalyl acetate on the pain experienced by young adults with postherpetic neuralgia.

## Conclusions

Collectively, the findings of this study of patients with postherpetic neuralgia showed that inhalation of lavender oil or either of its main constituents (linalool and linalyl acetate) reduced the quality, severity, and intensity of pain. These findings suggest that lavender oil, linalool, and linalyl acetate may be effective agents for reducing pain in patients with postherpetic neuralgia. Moreover, inhalation of lavender essential oil, linalool, or linalyl acetate by patients with postherpetic neuralgia is expected to allow physicians to reduce the dose or frequency of conventional pain killers. However, considering the above-mentioned limitations, more comprehensive studies are needed to generalize the findings of the present study.

## Data Availability

The datasets generated and/or analyzed during the current study are available from the corresponding author on reasonable request.

## Abbreviations

BDI: Beck Depression Inventory
GABA: γ-aminobutyric acid
PPI: Present pain intensity
SF-MPQ: Short form of the McGill Pain Questionnaire
TCAs: Tricyclic antidepressants
VAS: Visual analogue scale
